# Symptom burden, psychological distress, and health-related quality of life in cancer survivors with pelvic late radiation tissue injuries

**DOI:** 10.1007/s00520-021-06684-x

**Published:** 2021-11-15

**Authors:** Grete K. Velure, Bernd Müller, May Aa. Hauken

**Affiliations:** 1 Centre for Crisis Psychology, Faculty of Psychology, University of Bergen, Møllendalsbakken 9, N – 5009 Bergen, Norway; 2 Hyperbaric Medicine Unit, Department of Occupational Medicine, Haukeland University Hospital, Bergen, Norway

**Keywords:** Late effects, Long-term survivors, Pelvic malignancies, Pelvic radiotherapy, Psychological distress, Quality of life

## Abstract

**Purpose:**

Curative radiotherapy for cancer may lead to severe late radiation tissue injuries (LRTIs). However, limited knowledge exists about pelvic cancer survivors’ LRTI symptoms, distress, and health-related quality of life (HRQOL). We sought to assess the symptom burden, distress, and HRQOL in survivors with established pelvic LRTIs compared to norm populations and to investigate the relation between these factors.

**Methods:**

Cancer survivors referred for treatment of established pelvic LRTIs were recruited nationwide. LTRIs were assessed with the Expanded Prostate Cancer Index Composite (EPIC), psychological distress was assessed with the General Health Questionnaire (GHQ-12), and HRQOL was assessed with the European Organization for Research and Treatment of Cancer Quality of Life Questionnaire (EORCT-QLQ-C30).

**Results:**

A total of 107 participants (mean age 64, 53% men) were included. Compared to norms, participants reported more urinary (mean 68.7 vs. 89.5; *p* = 0.00; *d* = 1.4) and bowel symptoms (mean 62.5 vs. 92.4; *p* = 0.00; *d* = 2.7), increased psychological distress (mean 13.4 vs. 10.3; *p* = 0.00; *d* = 0.6), and overall poorer HRQOL (mean 54.9 vs. 71.2; *p* = 0.00; *d* = 0.7). Higher symptom burden and higher levels of psychological distress were associated with lower HRQOL (*r*^2^ = 46%), but psychological distress did not moderate the influence of symptoms on HRQOL.

**Conclusion:**

Cancer survivors with established pelvic LRTIs are highly burdened compared to norms. The association of the LRTI-related symptom burden with HRQOL is independent of the level of psychological distress. Both coping and treatment interventions are crucial to promoting long-term health and HRQOL.

**Trial registration:**

NCT03570229.

## Introduction

Annually, more than 34,000 Norwegians are diagnosed with cancer, where pelvic malignancies—including prostate, urological, bowel, and gynaecological malignancies—account for approximately 35% of all cases [1]. Radiotherapy is an important part of the multimodal curative treatment for pelvic cancers. However, as radiation also affects normal tissue, it may lead to radiation tissue injuries that can increase or persist for a long time and are often severe [2–6]. Adverse effects of radiotherapy on normal tissue leave approximately 5–15% of patients with late radiation tissue injuries (LRTIs) [7]. Pelvic LRTIs are characterized by tissue damage, fibrosis, hypoxia, and poor microcirculation affecting the bowel, urinary tract, genitalia, and pelvic bones [7]. Symptoms such as diarrhoea, faecal leakage, incontinence, haematuria, increased urinary/ bowel frequency, increased urinary/bowel urgency, and sexual dysfunction are documented in survivors of rectal, anal, urological, prostate, and gynaecological malignancies [8–10]. These cancer survivors experience severe symptom burden, especially related to bowel symptoms, although symptoms often decrease over time [10, 11]. On the other hand, pelvic LRTI symptoms are often underdiagnosed and are often interpreted as symptoms related to aging, and thus, only a minority are referred to follow-up and treatment [2, 7]. Furthermore, the treatment options for pelvic LRTIs are limited and mostly focused on symptom relief [12].

Late effects from cancer and cancer treatment, especially radiotherapy, are associated with psychological distress. This includes emotional symptoms such as worry, sorrow, anxiety, and depression, where higher symptom burden predicts higher levels of distress across cancer diagnoses [13–16]. In addition to the symptom burden, it is crucial to have a focus on psychological distress because this may also impair health-related quality of life (HRQOL) and increase poor health behaviours, consumption of medical resources, and mortality [17, 18]. However, studies of psychological distress in survivors with pelvic LRTIs are very limited. Bergerot et al. [19] showed that patients with gynaecological and gastrointestinal cancers are in general at higher risk of psychological distress. Adams et al. [2] found that more severe pelvic LRTI symptoms across cancer types were associated with higher rates of depression but not with higher rates of anxiety.

It is well-established that late effects from cancer may affect all areas of cancer survivors’ lives [20]. Based on a bio-psychological view of health, HRQOL is defined as an individual, subjective, multidimensional, and dynamic concept and is reckoned as an important outcome of cancer survivors’ perceived health and well-being after cancer treatment [21, 22]. HRQOL theory posits that challenges and strengths within each dimension will contribute to the individuals’ overall HRQOL [23]. This implies that distress from pelvic LRTI symptoms may negatively influence the different dimensions of the cancer survivors’ HRQOL and overall HRQOL. Consequently, improvements in LRTI symptoms or any other HRQOL dimension may positively influence HRQOL. Thus, HRQOL may give a holistic picture of the cancer survivors’ perceived health and overall well-being. Previous studies indicate that pelvic LRTIs across cancer types may severely impair the survivors’ HRQOL, where higher treatment toxicity and comorbidity after radiation as well as combinations of chemotherapy and radiotherapy seem to be important risk factors [24–26]. Nevertheless, there is a lack of studies focusing on the influence of pelvic LRTI symptoms on HRQOL.

Based on the outlined research and the theoretical framework, we have limited knowledge about the levels of symptom burden, distress, and HRQOL in cancer survivors with pelvic LRTI symptoms compared to norms. Furthermore, the relationship between pelvic LRTIs, psychological distress, and HRQOL remains unclear, including with respect to whether the degree of experienced psychological distress influences the symptoms’ relation with HRQOL.

This is important knowledge in planning effective treatment interventions, following up on survivorship, and promoting long-term health and HRQOL for survivors with pelvic LRTIs.

### Study aims

**Fig. 1:**
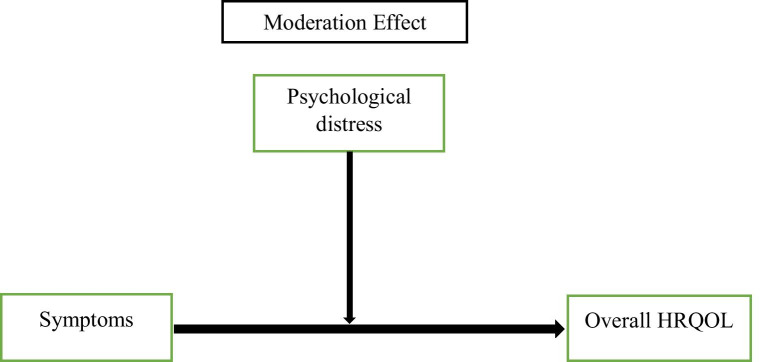
Conceptual framework. Abbreviations: HRQOL, health-related quality of life

The overall aim of this study was to explore symptoms, psychological distress, and HRQOL in cancer survivors with pelvic LRTIs and the relationship between these outcome variables. The conceptual framework is outlined in Fig. 1. More specifically, we aimed to:

1. Investigate pelvic LRTI symptoms, psychological distress, and HRQOL in cancer survivors compared to norm populations.
2. Study the influence of pelvic LRTI symptoms and psychological distress on HRQOL and investigate whether the relation between LRTI symptoms and HRQOL is moderated by psychological distress.

## Methods

### Study design

This study is a part of a prospective longitudinal study aiming to increase the knowledge about pelvic LRTIs in cancer survivors undergoing hyperbaric oxygen therapy (trial registration: ClinicalTrials.gov. Identifier: NCT03570229). The present study builds on baseline data assessed before hyperbaric oxygen therapy. Here, we used a descriptive cross-sectional study design with the purpose to identify potentially related factors, conveying more knowledge about the disease or condition, and illuminate areas for further study [27].

### Recruitment and eligibility criteria

The study sample was recruited from cancer survivors with pelvic LRTIs assigned to the Norwegian National Unit for Planned Hyperbaric Oxygen Treatment. Inclusion criteria were (a) pelvic radiation injury after intended curative radiation for pelvic cancer (prostate, gynaecological, urological, bowel, and bone cancers); (b) LRTI symptoms from the bowel, bladder, or pelvic area verified by endoscopy or radiology; (c) ≥ 6 months from completion of radiation therapy; and (d) age ≥ 18 years. Exclusion criteria were (a) severe physical and/or mental comorbidity representing a contraindication for hyperbaric oxygen therapy; (b) insufficient language skills to answer study questionnaires; (c) previous treatment with hyperbaric oxygen; and (d) signs of active cancer (or metastasis).

### Data collection

Data were collected from August 2018 to October 2020 by three self-reported questionnaires, which were sent by post before treatment start. The participants returned the questionnaires and written consent by post or when arriving at the hyperbaric unit for their first treatment.

- The Expanded Prostate Cancer Index Composite (EPIC) [28] was used to assess pelvic LRTI symptoms. This instrument is validated for prostate cancer, gynaecological malignancies [29], and complications from radiotherapy, and measures urinary and bowel symptoms based on the past 4 weeks. Items are scored on Likert scales (0–4, 1–3, 1–4, and 1–5) that are transformed into a 0–100 score. Results are presented as a total score for urinary and bowel symptoms based on the means of all items, as well as urinary subscales (function, bother, incontinence, irritation/ obstruction) and a bowel subscale (function, bother). A lower score indicates more severe symptoms in all domains. These domains are shown to be valid, reliable, and sensitive instrument to assess urinary and bowel toxicity (Cronbach’s alpha EPIC urinary/ bowel total scores ≥ 0.82) [28, 29].
- Psychological distress was assessed by the General Health Questionnaire (GHQ-12) [30]. This instrument consists of 12 items scored on a four-point Likert-type scale (0–3) to assess distress severity over the past 2 weeks. All items are summed to a score ranging from 0 to 36. A higher score indicates more symptoms of psychological distress [30]. The instrument is widely used to measure the mental distress, showing generally high validity, sensitivity, and specificity (Cronbach’s alpha range between 0.82 and 0.86) [30, 31].
- HRQOL was measured with the European Organization for Research and Treatment of Cancer (EORTC) quality of life questionnaire (QLQ-C30, version 3.0) [22]. This instrument consists of 30 questions, where items are scored on Likert scales (1–4, 1–7). All items are transformed into a 0–100 score and combined into five functional scales, nine symptom scales, and an overall HRQOL scale [32]. For functional scales and overall HRQOL, a high score reflects a high level of functional capacity. Conversely, high scores on the symptom scales represent a high symptom burden associated with poor HRQOL. This instrument is widely used both internationally and nationally with documented robust psychometric properties shown to be a reliable and valid measure of the HRQOL of cancer patients (Cronbach’s alpha range between 0.80 and 0.90 for most multi-item scales and single items) [33].

To ensure acceptable work load and that the questions were understandable and relevant, four cancer survivors with pelvic LRTIs, previously treated with hyperbaric oxygen therapy, and not participating in the study, tested and gave positive feedback about the questionnaires.

### Statistical analysis

Statistical analysis was conducted using IBM SPSS Statistics for Windows version 26 [34]. All variables were normally distributed as determined by Q-Q plots, skewness, and kurtosis. Internal consistency, measured by Cronbach’s alpha, was high for all instruments (*α* = 0.80–0.91). The few missing values were handled according to the respective questionnaires’ manuals [30, 32, 35].

Descriptive statistics were used for demographic and medical variables. *Z-*tests were used to explore the differences between the participants’ mean scores and the mean scores in the norm populations. The effect size of the differences was calculated using Cohen’s *d* and judged as small (*d* = 0.2), medium (*d* = 0.5), large (*d* = 0.8,) or very large (*d* = 1.3) [36].

The EPIC mean scores (urinary/bowel total scores) were compared with controls without prostate cancer (*N* = 112) [37]. The GHQ-12 mean scores were compared with a sample consisting of married/cohabiting students (*N* = 1750), published by Nerdrum et al. [38]. Mean scores of HRQOL were compared to the EORTC reference values of a general European population (*N* = 7802) [33]. The manual suggests changes of clinical significance to be 8 endpoints in overall HRQOL as a primary outcome [33]. Using the ‘true value’ (mean score = 61.4/SD = 24.7) on overall HRQOL, the estimated mean will be 68.3 for the participants. Based on a two-sided significance level of *α* = 0.05 and a power of 80% (*β* = 0.20), we needed a sample size of 81. With estimated 20% dropout, the warranted samples were 101 participants.

Background variables as age, gender, type of cancer treatment, and radiation-related variables were regarded as important variables, and all outcome variables were controlled against these using the independent-samples *t*-test. Regression analysis was used to assess the influence of age and clinical variables (cancer site, time since treatment, and radiation dose) [39]. Correlation analysis, using Pearson’s *r* and explained variance (*r*^2^), was performed between pelvic LRTI symptoms, psychological distress, and overall HRQOL. Multiple linear regression analysis was carried out to explore the relationship between pelvic LRTI symptoms, psychological distress, and overall HRQOL (model 1) [40]. A moderation analysis was conducted to examine the influence of psychological distress (the moderator) on the association of pelvic LRTI symptoms with overall HRQOL, by adding the product of psychological distress and pelvic LRTI symptoms to the multiple regression analyses (model 2) [40]. For all analyses, a two-tailed *P*-value < 0.05 was set as the significance level.

### Ethical considerations

The study was approved by the Regional Committee of Medical and Health Research Ethics, Northern Norway. (ID-number: 2018/706) and was conducted in compliance with the Declaration of Helsinki and the requirement for data processing and handling of the data [41]. The participants received written information about the study that participation in the study was voluntary, that all data would be treated confidentially, that they could withdraw from the study at any time, and that data could be deleted on request. All participants gave written consent.

## Results

### Study population

In total, 129 survivors met the eligibility criteria, and 107 participants were included in the study. Non-participation was related to declining to participate (*n* = 11), withdrawal from treatment (*n* = 6), and previous hyperbaric oxygen therapy (*n* = 5). The participants’ mean age was 64 years, slightly more were men (53.3%), and the majority were married/cohabiting (72%). Most participants had a college or university education, but only a few worked full or part time. The majority had pelvic LRTI injuries from prostate or gynaecological cancers (88%), and the mean time since radiation was 70.5 months. Demographic and medical characteristics are outlined further in Table 1.

**Table 1:** Demographic and medical variables

	*n* (%)
Gender	
Female	50 (46.7)
Male	57 (53.3)
Age, years [mean (SD, range)]	64 (12, 32–84)
Education	
Primary/high school	22 (20.5)
College/university	85 (79.5)
Work status	
Full time/part time employment	19 (17.7)
Sick leave/disability pension/retired	88 (82.3)
Civil status	
Single	30 (28.0)
Married/cohabiting	77 (72.0)
Children under 18 years of age	
Yes	13 (12.1)
No	94 (87.9)
Medical characteristics	
Cancer site	
Rectum/anus	13 (12.1)
Prostate	56 (52.4)
Gynaecological	38 (35.5)
Referral diagnosis	
Proctitis	45 (42.1)
Cystitis	39 (36.4)
Proctitis and cystitis	9 (9.4)
Osteoradionecrosis pelvis	11 (10.3)
Wound/fistula	3 (2.8)
Type of cancer treatment	
Chemotherapy and radiation	39 (36.4)
Radiation only	68 (63.6)
Types of radiation	
External only	77 (72.0)
External and internal	30 (28.0)
Radiation dose, Gy [range]	
External	35.0–100.0
Internal	7.0–75.0
Months since radiation [mean (SD, range)]	70.48 (78.32, 11–511)

Abbreviations: *Gy*, Gray; *SD*, standard deviation. Numbers are number of participants (% of total) if not specified otherwise

### Pelvic LRTI symptoms, psychological distress, and HRQOL

Addressing our first study aim, we found that cancer survivors with pelvic LRTIs experienced considerably more symptoms, psychological distress, and impaired overall HRQOL than norms. Mean scores for LRTI symptoms, psychological distress, and HRQOL, as well as comparison with the respective norms, are presented in Table 2.

**Table 2:** Symptoms, psychological distress, and health-related quality of life compared to norm populations

EPIC	Study population *N* = 107	Controls without cancer^a^ *N* = 112	Study population vs. controls		
	Mean (SD)	Mean (SD)	Diff	*z/ P*	*d*
Urinary total	68.7 (18.0)	89.5 (11.2)	− 20.8	− 10.4/0.00	1.4
Urinary function	68.1 (27.9)	95.5 (9.5)	− 27.3	− 9.3/0.00	1.5
Urinary bother	69.0 (17.0)	85.2 (14.1)	− 16.2	− 7.7/0.00	1.0
Bowel total	62.5 (13.6)	92.4 (8.7)	− 29.9	− 19.0/0.00	2.7
Bowel function	60.3 (18.0)	92.1 (8.5)	− 31.8	− 17.7/0.00	2.4
Bowel bother	64.5 (15.5)	92.8 (11.1)	− 28.3	− 15.7/0.00	2.1
GHQ-12		Cohabiting/married adults^b^ *N* = 1750	Study population vs. healthy adults		
Psychological distress	13.4 (5.5)	10.3 (4.9)	3.1	6.2/0.00	0.6
EORTC-QLQ-C30		General population^c^ *N* = 7802	Study population vs. general population		
Overall HRQOL	54.9 (22.6)	71.2 (22.4)	− 16.3	− 7.4/0.00	0.7
Physical function	69.1 (23.7)	89.8 (16.2)	− 20.7	− 9.0/0.00	1.2
Role function	59.9 (35.7)	84.7 (25.4)	− 24.8	− 7.2/0.00	0.9
Emotional function	73.6 (23.8)	76.3 (22.8)	− 2.7	− 1.2/0.22	0.1
Cognitive function	72.0 (27.5)	86.1 (20.0)	− 14.1	− 5.2/0.00	0.7
Social function	48.3 (32.1)	87.5 (22.9)	− 39.2	− 12/0.00	1.7
Fatigue	49.8 (28.5)	24.1 (24.0)	− 25.7	9.2/0.00	1.1
Nausea and vomiting	9.7 (16.0)	3.7 (11.7)	6.0	4.0/0.00	0.5
Pain	39.6 (32.6)	20.9 (27.6)	18.7	5.8/0.00	0.7
Dyspnoea	26.5 (29.3)	11.8 (22.8)	14.7	5.3/0.00	0.6
Insomnia	47.1 (32.7)	21.8 (29.7)	25.3	7.9/0.00	0.9
Appetite loss	16.0 (25.0)	6.7 (18.3)	9.5	4.0/0.00	0.5
Constipation	28.6 (32.7)	6.7 (18.4)	21.9	7.1/0.00	1.2
Diarrhoea	50.5 (35.5)	7.0 (18.0)	43.5	12.8/0.00	2.3
Financial difficulties	20.6 (32.9)	9.5 (23.3)	11.1	3.5/0.00	0.5

Abbreviations: *d*, effect size, judged as small (*d* ≥ 0.2), medium (*d* ≥ 0.5), large (*d* ≥ 0.8) or very large (*d* ≥ 1.3); *EORTC-QLQ-C30*, European Organization for Research and Treatment of Cancer Quality of Life Questionnaire; *EPIC*, The Expanded Prostate Cancer Index Composite; *GHQ-12*, General Health Questionnaire; *Overall HRQOL*, overall health-related quality of life; *P*, statistically significance difference < 0.05; *SD*, standard deviation; *z score*, provided by *z* test

Norm populations: ^a^EPIC, control population [37]; ^b^GHQ-12, studied by Nerdrum et al. [38]; ^c^EORTC-QLQ-C30, reference value manual [33]

Compared to norms, the participants reported a higher symptom burden on EPIC bowel and urinary total scales and on all subscales, mostly with very large effect sizes. Women reported more bowel total symptoms than men (mean 58.6 vs. 65.7, *P* = 0.00). Participants treated with both chemotherapy and radiation reported more total bowel symptoms than participants treated with radiation only (mean 58.8 vs. 65.0, *P* = 0.02). The participants also scored higher on psychological distress than the norm, with a medium-size difference (*P* = 0.00).

**Fig. 2:**
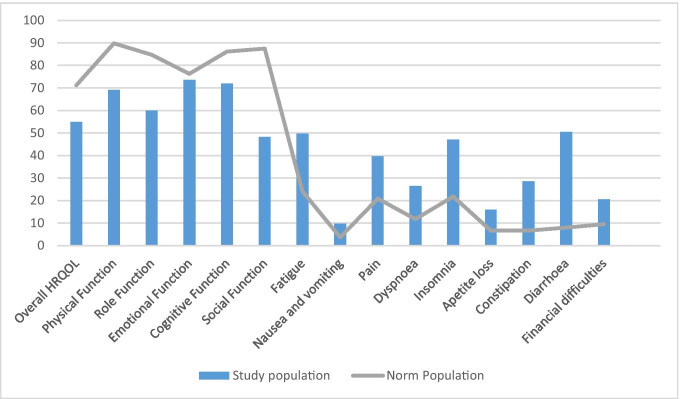
EORTC-QLQ-C30 mean scores compared to norm population^a^. Abbreviations: EORTC-QLQ-C30, European Organization for Research and Treatment of Cancer Quality of Life Questionnaire; HRQOL, health-related quality of life. For functional scales and overall HRQOL, a high score reflects a high level of functional capacity. High scores on the symptom scales represent a high symptom burden associated with poor HRQOL. Norm population: ^a^EORTC-QLQ-C30, reference values manual [33]

The participants scored lower than the general population on overall HRQOL and on all of the subdimensions, except for emotional function. The largest differences were observed for social function, physical function, and role function with large or very large effect sizes. The participants scored significantly higher than the norm on all symptom scales, with very large or large effect sizes for diarrhoea, constipation, fatigue, and insomnia as illustrated in Fig. 2. Participants working full or part time scored higher on overall HRQOL than those not working (*F* = 11,50/*P* = 0.00).

Regression analysis showed no association between EPIC urinary/ bowel symptoms, psychological distress, or overall HRQOL and age, cancer site, time since treatment, or radiation dose.

### The influence of pelvic LRTI symptoms and psychological distress on HRQOL

Addressing the first part of our second study aim, we tested the influence of pelvic LRTI symptoms and psychological distress on HRQOL. LRTI symptoms were positively correlated with HRQOL, meaning that a higher symptom burden is associated with lower HRQOL. The strongest negative correlation was found between psychological distress and HRQOL, predicting that a higher level of distress is associated with a lower level of HRQOL. Urinary and bowel symptoms were also negatively correlated with psychological distress (Table 3).

**Table 3:** Correlation analysis between HRQOL, symptoms, and distress

Dependent variable	Correlation	Urinary total	Bowel total	Psychological distress
Overall HRQOL	Pearson *r* *P*	0.7 0.00	0.28 0.00	− 0.55 0.00
Psychological distress	Pearson *r* *P*	− 0.19 0.03	− 0.24 0.01	

Abbreviations: *HRQOL*, health-related quality of life; *P*, statistical significance

The multiple linear regression analysis (model 1) showed that LRTI symptoms and psychological distress together explained 46.8% of the variance of overall HRQOL. Addressing the second part of our second study aim, we tested the moderation effect of psychological distress. Despite the high correlation of psychological distress with overall HRQOL, the moderation analysis (model 2) showed that psychological distress did not moderate the association of the severity of LRTI symptoms with HRQOL. This means that the influence of LRTI symptoms on HRQOL is independent of the level of distress (Table 4).

**Table 4:** Multiple regression models for overall health-related quality of life scores

Overall HRQOL^a^	*B*	SE *B*	*β/*(*P*)	Multicollinearity	*r*^2^
*Model 1*					
Constant	51.30	11.92			0.468
Urinary total	0.30	0.09	0.24 (0.00)	0.95	
Bowel total	0.19	0.13	0.12/(0.13)	0.93	
Psychological distress	− 2.20	0.31	− 0.55/(0.00)	0.91	
*Model 2*					
Constant	28.55	25.70			0.473
Urinary total	0.45	0.26	0.36/(0.08)	0.13	
Bowel total	0.40	0.33	0.24/(0.23)	0.14	
Psychological distress	− 0.44	1.80	− 0.11/(0.80)	0.03	
Psychological distress × urinary total	− 0.01	0.02	− 0.21/(0.53)	0.05	
Psychological distress × bowel total	− 0.02	0.03	− 0.25/(0.50)	0.04	

Abbreviations: *B*, unstandardized regression coefficient; *HRQOL*, health-related quality of life; *Model*, ʽenterʼ method in SPSS statistics; *Multicolinearity*, tolerance factor; *P*, significance level; *r*^2^, explained variance; SE *B*, standard error of the coefficient; *β*, standardized coefficient. ^a^Dependent variable

## Discussion

To our knowledge, this is the first study to focus on the level of symptom burden, distress, and HRQOL compared to norm as well as the interaction between these variables in cancer survivors with pelvic LRTIs.

It is well-known that radiotherapy to the pelvic area may cause severe side effects [3, 8, 9]. However, studies on long-term pelvic LRTIs are sparse, and thus, the present study contributes to improve this knowledge. At a mean time of nearly 6 years from the end of radiotherapy, the participants reported significantly higher levels of LRTI symptoms compared to a norm population. Similar results have been shown in previous studies [25, 42]. No differences in the symptom profile across cancer types, age, or time since treatment were found, except that women had higher bowel impairment. This aligns with other studies indicating that survivors after gynaecological cancer are especially affected by bowel symptoms [3, 10]. This may be explained by an objective increased affection of bowel function based on anatomic gender differences, or the more frequent application of brachytherapy and multimodal treatment in women compared to men [11]. Furthermore, bowel symptoms such as faecal urgency or leakage may be particular embarrassing and might poorly correspond with feelings of femininity in terms of body image, attractiveness, and sexuality [3].

The participants reported moderately more psychological distress than norms. This supports earlier findings of high levels of anxiety, depression, and impaired mental health among survivors treated for different pelvic malignancies [2, 26]. To the best of our knowledge, this is the first study reporting psychological distress and the effect sizes of differences in cancer survivors with established pelvic LRTIs compared to norms. Unlike other studies, no interaction between age and psychological distress was found [26, 43]. Compared to norms, the participants reported a large impairment in overall HRQOL and in all the functional subdimensions, except for emotional function, as well as a high symptom burden for fatigue, insomnia, and pain. Corresponding studies on the long-term HRQOL of survivors with pelvic malignancies report slightly better overall HRQOL [9, 25], mainly explained by complete disease remission and the decline of symptoms over time [11, 24]. Here, an obvious explanation may be that our participants represent a selected sample with established LRTIs where the symptoms had not declined over time. Overall, these findings indicate that all areas of the participants’ lives are negatively affected. However, an interesting finding is that their emotional function was comparable to the norm population. One explanation may be that the participants have adapted and developed several coping strategies related to their pelvic LRTIs. Another explanation may be that they were about to start hyperbaric oxygen therapy and consequently had hope for a positive outcome, which is an important factor for coping and for HRQOL [44].

More than just the single variables of symptom burden, distress, and HRQOL, the interactions found between these variables are important. First, the results revealed a strong correlation between LRTI symptoms and HRQOL, confirming previous research on symptom burden as a risk factor for impaired HRQOL [6, 11, 24–26]. It is worrisome that these patients often are underdiagnosed and undertreated, although the symptom burden severely impairs HRQOL [2, 7].

Second, the participants’ elevated levels of psychological distress also impaired their HRQOL negatively. This may be interpreted as a normal reaction to the everyday burden of living with LRTIs. However, an interesting finding is that the pelvic LRTI symptoms affected HRQOL regardless of the level of psychological distress. This indicates that the symptom burden is a strong predictor for impaired HRQOL in cancer survivors with pelvic LRTIs, which aligns with previous research suggesting that more cancer survivors have reduced HRQOL as a result of physical impairments rather than psychological impairments [18]. Third, the fact that psychological distress did not moderate the connection between symptom burden and HRQOL might have several relevant explanations, such as elevated levels of psychological distress before cancer treatment, going through a life-threatening diagnosis and treatment, or anxiety about cancer recurrence [16, 45]. Another explanation for the elevated distress may be related to hyperbaric oxygen therapy the participants were about to start, as this represents a new, highly technological, and unknown treatment for most patients. On the other hand, the significant association between HRQOL and psychological distress, as well as the symptoms’ significant correlation with psychological distress, indicate the importance of screening and identifying survivors in need of psychological distress interventions in addition to pelvic LRTI symptom management.

Overall, this study’s results underline the complexity and interactions between LTRI symptoms, psychological distress, and HRQOL and the importance of a bio-psychological or holistic view in screening, survivorship follow-up, and interventions.

### Clinical implications

The results documenting a high symptom burden, elevated distress, and impaired HRQOL raise several implications for clinical practice and further research. First, the results indicate that several cancer survivors with pelvic LRTIs have significantly impaired HRQOL and debilitating symptoms several years after radiation. Consequently, there seems to be a need for increased competence and education of healthcare professionals about LRTIs. Second, cancer survivors with pelvic cancers should be informed about LRTIs as a possible late effect from radiation, and which symptoms to be aware of. Third, systematic assessment of pelvic LRTI symptoms and HRQOL after radiation should be part of routine follow-up, whereby impairment should be addressed with proper symptom management and educating survivors in adequate coping skills (e.g. hyperbaric oxygen therapy, rehabilitation programme). Fourth, with persisting symptoms, early diagnosis of established pelvic LRTIs should be confirmed by objective measures and available treatment options as, for example, hyperbaric oxygen therapy should be considered. Finally, overall, nurses play a crucial role in supporting cancer survivors with pelvic LRTIs in all these means, especially by encouraging them to express their needs, screening for LTRI symptoms, and promoting coping and effective treatment interventions to decrease the symptom burden. Furthermore, nurses should have a holistic approach and screen for impaired HRQOL, acknowledging that other factors than the LRTI symptoms may be a source of increased distress. More research in this field is highly needed, especially related to the survivorship follow-up, effects of available treatment options, and rehabilitation programmes.

### Strengths

Study strengths are the inclusion of a relatively large and national cohort of both men and women with a range of clinically significant and objectively verified pelvic LRTIs.

Symptoms, distress, and HRQOL were evaluated with validated, well-recognized instruments, and the outcomes were compared to established norms. Furthermore, high survey completion rates strengthen the study. However, the focus on a selective population referred to hyperbaric oxygen therapy may limit the generalization of the findings.

## Conclusion

Cancer survivors with established LRTIs reported a severe symptom burden, moderate levels of psychological distress, and highly impaired HRQOL compared to norms several years after radiation. To improve HRQOL, treatment of pelvic LRTI symptoms and interventions related to coping are of great importance.

## Data Availability

De-identified data will be available from the study leader on reasonable request after the end of the project.
